# Editorial: Molecular Adaptations of Vibrionaceae to Changing Environments, Volume II

**DOI:** 10.3389/fmicb.2022.863038

**Published:** 2022-03-07

**Authors:** Jens A. Hammerl, Jennifer M. Ritchie, Francesca Leoni, Swapan Banerjee, Eckhard Strauch

**Affiliations:** ^1^Consultant Laboratory for Vibrio spp. in Food, Department Biological Safety, German Federal Institute for Risk Assessment, Berlin, Germany; ^2^Department of Microbial Sciences, Faculty of Health and Medical Sciences, University of Surrey, Guildford, United Kingdom; ^3^National Reference Laboratory for Control of Bacteriological Contamination of Bivalve Molluscs, Istituto Zooprofilattico Sperimentale dell'Umbria e delle Marche “Togo Rosati”, Ancona, Italy; ^4^Vibrio Laboratory, Bureau of Microbial Hazards, Health Canada, Ottawa, ON, Canada

**Keywords:** vibrio, pathogenicity, virulence, lifestyle, adaption, diversity

The family Vibrionaceae contains a broad spectrum of bacterial genera that are ubiquitous in aquatic ecosystems worldwide (Jiang et al., 2022). Due to their presence in different environments (i.e., water, sediments, aquatic organisms), the bacteria need to adapt to the prevailing conditions found in specific habitats (Sakib et al., 2018; Ceccarelli et al., 2019). These can include a mammalian host as some members of the Vibrionaceae can cause severe infections in animals and/or humans (Baker-Austin et al., 2018). In the face of global climate change, our focus on these organisms is heightened and a deeper understanding of the pathways and mechanisms involved in environmental/host adaption and pathogenicity is overdue. Detection of tropically predominant *V. cholerae* in cold temperate regions (Huehn et al., 2014; Banerjee et al., 2018) provides important subject matter for studies determining the evolutionary changes that prompted adaptation to this environment.

This Research Topic brings together fourteen articles featuring recent discoveries on the biology of *Vibrio* species (including a single study on *Photobacterium*) covering (i) epidemiological aspects, (ii) their adaption to specific ecosystems, (iii) changes in their lifestyle caused by prevailing conditions or stress responses, as well as (iv) their antimicrobial susceptibility and mobile genetic elements involved in horizontal gene transfer. The articles summarize important aspects, which are relevant for risk evaluation of species with pathogenic potential, but also to gain a deeper knowledge of their phylogenetic relationships, lifestyles, and conditions leading to phenotypic changes. The Research Topic further includes three comprehensive literature reviews providing valuable overviews about specific pathogenic species and mechanisms causing their pathogenicity.

As experimentally determined in two studies, the water of lakes comprise important reservoirs for endemic-occurring O1 (Lake Victoria, Uganda) and non-O1/-O139 *V. cholerae* (lake Neusiedler See, Austria). In general, such natural water reservoirs exhibit diverse *V. cholerae* populations that can be associated with (cross)-regional pandemics (*V. cholerae* O1) or individual human infections (*V. cholerae* non-O1/-O139). As shown for Lake Victoria (Hounmanou et al.), the investigated *V. cholerae* O1 isolates are closely related to pandemic isolates of adjacent countries (Kenya, Tanzania etc.). Thus, they represent a natural reservoir (i.e., water, plankton, fish) for long-term persistence and spatial distribution of the bacteria as well as a “melting point” for the development of novel *V. cholerae* pathotypes. In Austria, the lake Neusiedler See and other lakes possess endemic non-O1/-O139 *V. cholera* populations (Lepuschitz et al.), which are sporadically associated with human infections due to direct water contact (i.e. via recreational activities like bathing). Based on the type of infection, isolate characterization focused on their phylogenetic relationship and antimicrobial susceptibility. Despite a broad genetic diversity, the Neusiedler See-isolates exhibited only a few antimicrobial resistances of clinical importance and pose only a low impact as natural drivers for their spread. The study of Hounmanou et al. strengthens the hypothesis that fish are a common source for non-toxigenic and toxigenic *V. cholerae*. The investigation of the group confirmed the colonization of the intestines of tilapia (*Oreochromis niloticus*) and showed their transmission to naïve fishes of the same type. Based on these results a long-time persistence of *V. cholerae* in aquatic animals (i.e., fish, marine mammals), as well as a transmission to other fish species or aquatic animals can be expected. The study of Hussain et al. provides insights into the impact of the *V. cholerae* type VI secretion system (T6SS) operons on the environmental lifestyle and adaptation of the species. In fact, the investigation of T6SS-mediated dynamics of bacterial competition within an environmental North American *V. cholerae* population reveals the occurrence of diverse strain-specific repertoires of cytotoxic T6SS effector and immunity genes in the *V. cholerae* population. Competition experiments usually lead to “stalemates” between isolates of different T6SS-based configurations, but outcomes can be substantially affected by horizontally acquired effector and immunity genes. The authors find a temperature-dependent outcome in T6SS competition leading to the better adaption of environmental isolates under native conditions than pathogenic strains. Thus, T6SS-based competition can affect the fitness of *V. cholerae* populations and preserve their intraspecies diversity. As mentioned above, various studies deal with diverse bacterial populations, which are adapted to specific ecosystems. Furthermore, continuous genetic flux, as well as the evolution of specific clones as a consequence of lateral gene transfer, can be only evaluated with adequate typing methods. To distinguish the genetic diversity and virulence patterns of *V. cholerae* O1/O139 isolates, Kanampalliwar and Singh provided a new five-locus-based multilocus sequence typing (MLST) scheme, which can be helpful for isolate dissection without using next-generation sequencing-based methods (i.e., cgMLST, wgMLST). The new MLST scheme further offers a higher resolution on the genomic diversity than the previously broadly used macrorestriction analysis (PFGE). Organisms associated with aquatic environments can serve as “natural bioreactors” affecting the adaption and evolution of *Vibrio* species. The embedment of bacteria into phagosomes of eukaryotic cells is associated with systematic exposure to digestive processes (i.e., low pH, antimicrobial peptides, reactive oxygen/nitrogen species, proteolytic enzymes, and essential metal ions), which can be overcome by *V. cholerae* and *V. harveyi* through the acquisition or development of specific factors. As these factors play an important role in shaping the infectious and pathogenic potential of *Vibrio* spp., the review of Espinoza-Vergara et al. outlines current knowledge on the strategies and prevailing factors/mechanisms enabling the pathogenicity of *V. cholerae* and other important pathogens. Bueno et al. present a review on the current knowledge of the complex life cycle of *V. cholerae* and metabolic pathways involved in different environmental and infective stages. The authors summarize data on the metabolic plasticity that allows *V. cholerae* to persist under low oxygen concentrations and its impact on environmental and host adaptation. Another detailed literature summary on adaptations of *V. parahaemolyticus* to stress was provided by Pazhani et al. This review provides important insights into the main regulatory pathways of the response to different stresses that influence its survival, gut colonization, and virulence. This information contributes to a better understanding of the bacterium and its potential for disease development in humans and animals. Novel experimental data on the survival, morphological changes, and virulence gene expression of *V. parahaemolyticus* under digestive-like conditions were presented by Wang et al. Here, *V. parahaemolyticus* was shown to survive digestive fluids used to simulate the oral and stomach passage conditions found in humans. Within the gastric system, an increase in pH activates the bacterial defense/escape mechanism, promoting their survival and pathogenesis progression. The impact of mobile genetic elements and their horizontal transmission was demonstrated by Zheng et al. who provide insights into the genome of two multi-drug resistance plasmids from foodborne *V. parahaemolyticus* isolates. Remarkably, the genetic basis of both plasmids is closely related to an IncC plasmid from *Aeromonas hydrophila*, revealing that horizontal transmission of resistance plasmids among aquatic bacteria is common. Nevertheless, the role played by the environment is not sufficiently investigated. With the expected future antimicrobial crisis, more comprehensive information and surveillance data on the impact/interaction of aquatic organisms, and the spread of resistance are required.

The presence of a multifactorial system regulating disease development and severity was confirmed by transcriptomic analysis on *V. vulnificus* by Hernández-Cabanyero et al. The authors outline the impact of prevailing temperature, salt, and serum levels on the expression of virulence factors involved in specific traits (i.e. host colonization, disease development). It has been shown that temperature is important for the infectivity of this pathogen and that it needs to be considered in the context of global warming. In the study of Kumar et al., the suitability of a biofloc system using *Litopenaeus vannamei* as a promising aquaculture technology was shown. The biofloc system maintains water quality by recycling nutrients and improves the health status and resistance of shrimps to microbial infection. Surprisingly, the pathogen, an acute hepatopancreatic necrosis disease (AHPND)-causing *V. parahaemolyticus* (M0904) strain, switches its lifestyle from free-living virulent planktonic to a non-virulent biofilm phenotype in the biofloc setting. Thus, future intervention strategies may focus on influencing the phenotype of the pathogenic *Vibrio* instead of trying to eliminate the organism. Another interesting observation on the co-evolutionary interactions of *V. splendidus*-clade isolates in natural Pacific oyster beds is presented by Wegner et al. The researchers linked the presence of two genes (OG1907 and OG3159) to host-specific virulence patterns based on genotype-by-genotype interactions between oyster larvae and their sympatric *Vibrio* communities. The genes appear to act as a genetic switch by forcing coevolution between *Vibrio* and oysters and may thus be important factors in the dynamics of the disease. Lages et al. performed studies on *V. anguillarum*, which is an important fish pathogen. The research indicates that the disease severity caused by *V. anguillarum* on cold- and warm-water-adapted fish species is multifactorial. As the expression of most virulence factors depends on the prevailing temperature conditions and iron levels, the pathogenicity of this species can substantially vary among the affected fish species and in different aquatic ecosystems. For the preparation of suitable fish vaccines against *Vibrio*, these observations need to be taken into consideration. In a second paper by Lages et al., new light was shed on the regulation of the high-pathogenicity island *irp*-HPI, especially on the expression of piscibactin genes, of the fish pathogen *V. anguillarum*. The researchers found that temperature-dependent expression of *irp*-HPI was associated with the combined effect of the HPI-AraC-like transcriptional activator (PbtA) and other genomic-associated regulators. Overall, the modulation of piscibactin gene expression appears to influence the regulation of various other virulence factors depending on the prevailing temperatures. The study of Zhou et al. reports on the analysis of the genetic diversity of phage-resistant *V. alginolyticus* strains. Experimental dissection revealed that the investigated mutant strains were genetically and phenotypically different. Sequence comparisons with coevolved *V. alginolyticus* revealed single nucleotide variations and insertions/indels, which were mainly identified in genes involved in the synthesis of flagella and extracellular polysaccharide (EPS). Furthermore, alterations are also found in tRNA/rRNA genes as well as in *nrdA*, a gene affecting the mutation rate of the organism. Thus, stress caused by phage infections represents an important factor for the genomic evolution of *V. alginolyticus*.

One study focuses on *Photobacterium damselae* subsp. *damselae*, which can affect humans. The analysis of multi-omics results by Matanza and Osorio provides evidence that the opportunistic nature of the bacterium and the genetic configuration of specific clones are responsible for human infections. Surprisingly, temperature conditions in humans seem to trigger a defensive strategy in the bacterium, evident by the upregulation of genes involved in defense mechanisms and downregulation of virulence genes.

This Research Topic is dedicated to improving our knowledge and understanding of the biology, ecology, and adaptation of Vibrionaceae to their natural environment and analyzing changes in response to host defense mechanisms when transitioning between different environments. The research papers include *Vibrio* from environmental, clinical, and food sources in accordance with the “One Health” concept. In summary, by analysis of prevalence, pathogenesis, virulence and multidrug resistance with emphasis on *Vibrio*-host interaction, the papers of this Research Topic can be exploited for an assessment of public health risks associated with these bacteria.

## Author Contributions

JH, JR, FL, SB, and ES conceptualized the Research Topic. JH and ES developed and drafted the editorial. JR, FL, and SB provided critical reviews. All authors edited the manuscript. All authors contributed to the article and approved the submitted version.

## Funding

The work of JH and ES was supported by the German Federal Institute for Risk Assessment (BfR) in the framework of the “Consultant Laboratory for *Vibrio* spp. in food” (BfR 45-009).

## Conflict of Interest

The authors declare that the research was conducted in the absence of any commercial or financial relationships that could be construed as a potential conflict of interest.

## Publisher's Note

All claims expressed in this article are solely those of the authors and do not necessarily represent those of their affiliated organizations, or those of the publisher, the editors and the reviewers. Any product that may be evaluated in this article, or claim that may be made by its manufacturer, is not guaranteed or endorsed by the publisher.
